# Increasing Length of the *Babesia* Season in New England in the Climate Change Era

**DOI:** 10.1093/ofid/ofaf458

**Published:** 2025-08-01

**Authors:** John J Ross, Narath Carlile, Kevin L Ard

**Affiliations:** Department of Medicine, Brigham and Women's Hospital, Boston, Massachusetts, USA; Department of Medicine, Brigham and Women's Hospital, Boston, Massachusetts, USA; Division of Infectious Diseases, Department of Medicine, Massachusetts General Hospital, Boston, Massachusetts, USA

**Keywords:** *Babesia microti*, babesiosis, climate change, seasonal variation, tick-borne diseases, vector-borne diseases

## Abstract

In a retrospective cohort of 1130 patients with babesiosis over a period of 31 years, there was a marked expansion of the *Babesia* season over time, with an increase of 0.33 months per year (CI .27 to .39) in a generalized linear model. The mean number of months with patients developing symptomatic infection with *Babesia microti* rose from 2.2 before 2000 to 9.2 after 2015. In the climate change era, babesiosis should be considered in the differential diagnosis of patients presenting with fever and anemia outside of peak summer months.

## Teaser

In a retrospective cohort of 1130 patients with babesiosis in New England over a period of 31 years, there was a marked expansion of the duration of the Babesia season over time. In the climate change era, babesiosis should be considered in the differential diagnosis of patients presenting with fever and anemia outside of peak summer months.

The incidence of tickborne disease has recently increased in the United States, as longer summers and milder winters have led to surges in tick populations [1]. The impact has been most marked in New England, where temperatures are rising faster than the global average, particularly in winter [2].

Babesiosis is a tickborne illness of special concern, given its high fatality rates in older and immunocompromised patients. Once restricted to coastal islands, babesiosis has become hyperendemic in New England. Between 2011 and 2019, the incidence of babesiosis increased by 193% in Massachusetts, 338% in Connecticut, 1422% in Maine, and 1602% in Vermont [3].

We examined 31 years of data at our institutions to see if more patients are developing symptomatic babesiosis outside of the peak summer months, which might lead to delayed diagnosis and worse outcomes.

## METHODS

We performed a retrospective chart review of adults (age ≥18 years) presenting with babesiosis as a primary diagnosis to 3 teaching hospitals in Boston, Massachusetts (Brigham and Women's Hospital, Brigham and Women's Faulkner Hospital, and Massachusetts General Hospital) between 1 May 1993 and 1 May 2024. Most of the patient population at these facilities is from Massachusetts, with a minority coming from adjacent New England states. Charts were identified via the Research Patient Data Registry, a data warehouse containing inpatient and outpatient records from multiple hospital systems [4], using International Classification of Diseases, Ninth Revision (ICD-9) codes 088.82 and Tenth Revision (ICD-10) codes B60.0.

Records were searched for the keywords “*Babesia*” and “babesiosis,” noting the month of onset of symptoms (Supplementary Table 1), confirmatory testing, and recent epidemiologic exposures. Month of symptom onset was used instead of month of diagnosis, as diagnosis might be significantly delayed in cases presenting in winter months. For the diagnosis of babesiosis, we required either a positive blood parasite smear or serum PCR; we excluded cases diagnosed by serology alone, as antibody testing does not reliably distinguish between active and resolved *Babesia* infection [5].

The study was approved by the Partners Institutional Review Board (protocol 2024P000553). Informed consent was waived due to the lack of use of identifiable health information and the logistical difficulties in obtaining informed consent in a retrospective chart review study. This study followed relevant STROBE reporting guidelines. R was used for statistical analysis (Version 4.4.2, R Foundation for Statistical Computing, Vienna, Austria).

## RESULTS

1975 patient records were reviewed. Of these, 845 cases were rejected. In 439 cases, there was inadequate documentation of confirmatory testing or onset of symptoms. 174 cases were rejected as being diagnosed by serology alone. Tests for babesiosis were negative in 104 cases. 58 cases of “chronic *Babesia*,” diagnosed by integrative medicine practitioners, were rejected because confirmatory testing was not available for review, or because of the use of nonstandard tests. 48 cases were rejected because the patients were <18 years at the time of diagnosis, and 19 cases were rejected as being possibly acquired outside New England. Two records were training accounts using fictitious patients, and another was rejected for miscoding (the patient had berylliosis, not babesiosis).

Of the 1130 cases, 986 were probably acquired in Massachusetts, 43 in New Hampshire, 25 in Rhode Island, 8 in Maine, 6 in Connecticut, and 1 in Vermont. 61 patients reported recent travel to or residence in 2 or more New England states.

In a generalized linear model, *Babesia* diagnoses showed a strong correlation with each unit increase in time (year), corresponding to an annual growth in cases of 14.2% (95% confidence interval [CI], 13.1%–15.2%). The model had similar results with the addition of state of *Babesia* acquisition as a fixed effect, with an annual rise in cases of 14.2% (95% CI, 13.2% to 15.3%).

**Figure 1.**
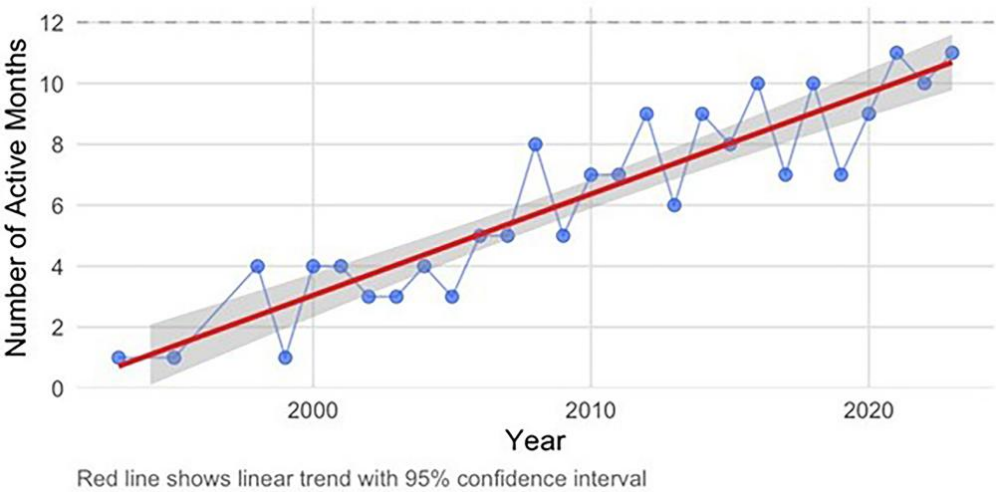
Expansion of the active season for the development of symptomatic babesiosis over time. In a generalized linear model, the number of months in which symptom onset occurs increased by 1 m every 3 y (0.33 m per year, CI .27 to .39). The mean number of months of the active season for *Babesia microti* rose from 2.2 before 2000 to 9.2 after 2015.

**Figure 2.**
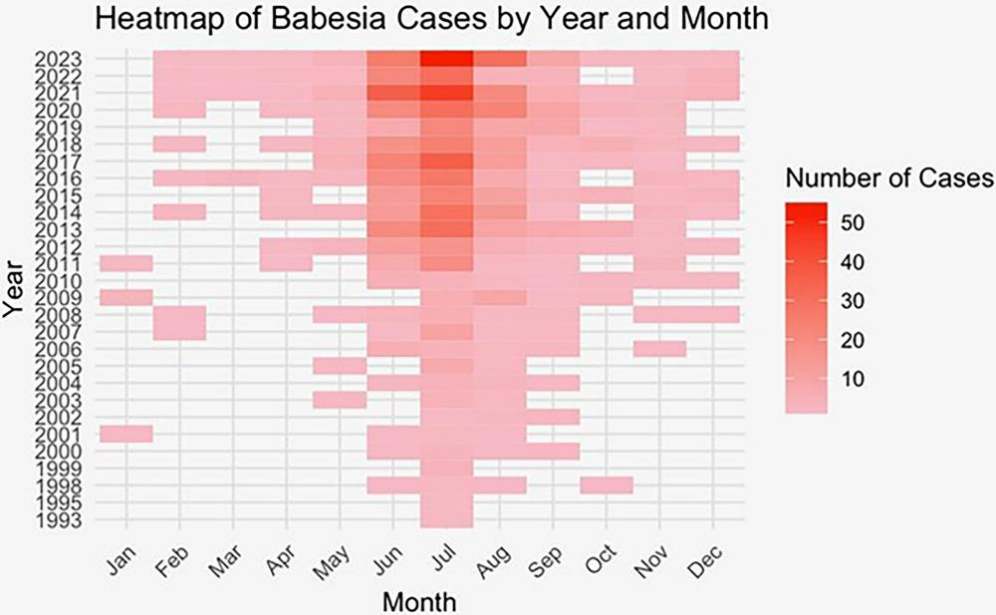
Heatmap of *Babesia* cases by year and month.

Cases continued to follow a marked seasonal pattern, with most patients reporting symptom onset in June and July, but the probability of cases in other months increased significantly over time (Figure 1). In a generalized linear model, the number of months in which symptom onset occurs increased by 1 month every 3 years (0.33 months per year, CI .27 to .39). The mean number of months of the active season for *Babesia microti* rose from 2.2 before 2000 to 9.2 after 2015. In 2023, it was 11 months long (Figure 2).

## DISCUSSION

We observed a striking expansion of both the annual caseload and the active season for *B. microti* over the past 31 years at our institutions, with an increase in cases occurring outside of summer months. Possible explanations include climatic, ecologic, and patient factors.

Between 1900 and 2010, New England experienced overall warming of 1.83°C. Most of this warming has taken place since the 1980s, corresponding to the surge in tick-borne diseases in the northeastern United States. This warming was most marked during the winter months, which saw an increase in minimum temperatures of 3.20°C [2]. These milder winter conditions likely improve survival of the primary vector of *B. microti*, the blacklegged tick (*Ixodes scapularis*), as well as its mammalian reservoirs. Severe winter cold is associated with high mortality in *Ixodes* ticks [6] and white-tailed deer [7], although the effects of cold snaps on *Ixodes* mortality are mitigated by the ability of ticks to shelter in protected microsites in the duff layer and soil [8].

Warming conditions also favor tick-human contact and *Babesia* transmission by accelerating tick life cycles and increasing tick activity outside of the traditional summer months. Warm temperatures shorten the time required for blacklegged ticks to mature, and increase the efficiency of pathogen transmission [9]. Adult blacklegged ticks are more active during winter warm spells [10], and warm spring conditions are associated with earlier activity of blacklegged tick nymphs [11]. Rising temperatures in late winter and early spring have been associated with an earlier start to the Lyme disease season [12, 13] and a higher incidence of Lyme disease in the Northeast [14]. The effects of climate on tick ecology are complex, and warming does not always favor disease transmission; hot and dry conditions lead to desiccation of ticks, and reduce questing activity and attachment to mammalian hosts [14, 15].

Disturbed ecosystem functioning may have played a role in the explosive growth of *Babesia* cases in recent decades. Tick-borne diseases in the northeastern United States are highly prevalent in fragmented suburban habitats with low mammalian biodiversity. The species that thrive in such areas, such as white-footed mice and eastern chipmunks, tend to “live fast and die young,” with high energy investment in growth and sexual reproduction, and low energy investment in immunity [16]. These characteristics make them excellent reservoirs and amplifying hosts for *B. microti* [17].

The increased use of immunosuppressive agents, particularly B-cell depleting therapies such as rituximab, which may unmask latent parasitemia, may be an additional reason for the development of symptoms outside of traditional peak months [18]. In immunocompetent hosts, asymptomatic, low-level parasitemia with *Babesia* may persist for more than 2 years if not treated [19]. The initiation of immunosuppression in these individuals may lead to symptomatic babesiosis in the absence of recent tick exposure, with the potential for severe and relapsing infection [18].

Factors not related to climate may also play a role in the rising incidence and expanded seasonality of *Babesia*, such as the increased availability of more sensitive nucleic acid amplification tests, increased patient and physician awareness, and mandatory reporting. However, our data show an expansion in the *Babesia* season prior to the onset of mandatory reporting, and the rising incidence of Lyme disease in the northeastern United States in one study was not related to increased awareness, as measured by tick-related Google searches [14].

A limitation of this study is the number of cases rejected for insufficient data, in part due to the limited information that could be extracted from legacy EHR systems. However, it is unlikely that this introduced a systematic bias with respect to *Babesia* seasonality.

In conclusion, we observed a significant expansion of the babesiosis season in the past 31 years, with the mean number of months per year with cases rising from 2.2 before 2000 to 9.2 after 2015. Possible explanations include diminished die-offs of blacklegged ticks during warmer winters, more rapid tick maturation in warm conditions, greater activity of adult ticks during winter warm spells, expansion of the active season for nymph forms into early spring and late fall, expanding populations and improving winter survival of tick hosts, and increased use of immunosuppressive agents such as anti-CD20 monoclonal antibodies in patients with latent *Babesia* infection. In endemic areas, babesiosis should be suspected in all patients presenting with fever and anemia, even during winter months.

## Supplementary Material

ofaf458_Supplementary_Data

## Supplementary Data

Supplementary materials are available at *Open Forum Infectious Diseases* online. Consisting of data provided by the authors to benefit the reader, the posted materials are not copyedited and are the sole responsibility of the authors, so questions or comments should be addressed to the corresponding author.
